# Hypoxia-Induced Alternative Splicing in Endothelial Cells

**DOI:** 10.1371/journal.pone.0042697

**Published:** 2012-08-02

**Authors:** Julia E. Weigand, Jes-Niels Boeckel, Pascal Gellert, Stefanie Dimmeler

**Affiliations:** 1 Institute for Cardiovascular Regeneration, Center of Molecular Medicine, Johann Wolfgang Goethe University Frankfurt, Frankfurt am Main, Germany; 2 Max-Planck Institute for Heart and Lung Research, Bad Nauheim, Germany; The John Curtin School of Medical Research, Australia

## Abstract

**Background:**

Adaptation to low oxygen by changing gene expression is vitally important for cell survival and tissue development. The sprouting of new blood vessels, initiated from endothelial cells, restores the oxygen supply of ischemic tissues. In contrast to the transcriptional response induced by hypoxia, which is mainly mediated by members of the HIF family, there are only few studies investigating alternative splicing events. Therefore, we performed an exon array for the genome-wide analysis of hypoxia-related changes of alternative splicing in endothelial cells.

**Methodology/Principal findings:**

Human umbilical vein endothelial cells (HUVECs) were incubated under hypoxic conditions (1% O_2_) for 48 h. Genome-wide transcript and exon expression levels were assessed using the Affymetrix GeneChip Human Exon 1.0 ST Array. We found altered expression of 294 genes after hypoxia treatment. Upregulated genes are highly enriched in glucose metabolism and angiogenesis related processes, whereas downregulated genes are mainly connected to cell cycle and DNA repair. Thus, gene expression patterns recapitulate known adaptations to low oxygen supply. Alternative splicing events, until now not related to hypoxia, are shown for nine genes: six which are implicated in angiogenesis-mediated cytoskeleton remodeling (*cask*, *itsn1*, *larp6*, *sptan1*, *tpm1* and *robo1*); one, which is involved in the synthesis of membrane-anchors (*pign*) and two universal regulators of gene expression (*cugbp1* and *max*).

**Conclusions/Significance:**

For the first time, this study investigates changes in splicing in the physiological response to hypoxia on a genome-wide scale. Nine alternative splicing events, until now not related to hypoxia, are reported, considerably expanding the information on splicing changes due to low oxygen supply. Therefore, this study provides further knowledge on hypoxia induced gene expression changes and presents new starting points to study the hypoxia adaptation of endothelial cells.

## Introduction

The sufficient supply of all cells of the body with oxygen is of vital importance and vessel growth is essential to promote blood supply after tissue ischemia. Particularly, the proliferation, migration and sprouting of endothelial cells from existing capillaries, a process termed angiogenesis, increases blood supply in ischemic tissues [1], [2]. On the other hand, angiogenesis is required for tumor growth and therefore is a target for anti-tumor therapies. The master regulator of the cellular response to hypoxia is the transcription factor HIF1 (hypoxia-inducible factor 1) [3]. Under normoxia the alpha subunit of HIF1 (HIF1A) is degraded via ubiquitin-dependent proteolysis. Hypoxia leads to the stabilization of the HIF1A protein and thereby initiates an orchestrated signaling response affecting the expression of direct and indirect target genes. The primary target genes of HIF1 are the cytokines VEGFA (vascular endothelial growth factor A) and EPO (erythropoietin). VEGFA and EPO stimulate angiogenesis, and EPO additionally enhances the production of red blood cells. Endothelial cells are the predominant target of VEGFA, because of their ability to grow towards a hypoxic region following a VEGFA gradient and thereby forming new blood vessels [4], [5].

The cellular signaling response to hypoxia needs to be tightly regulated, since already a small deviation of the balance between pro- and anti-angiogenic factors can cause several severe diseases such as ocular and inflammatory disorders. The “Von Hippel-Lindau” disease is caused by the non-degradation of HIF1A under normoxic conditions and results in the progressive growth of several cancer types such as hemangioblast or renal cell carcinoma upon excessive proangiogenic signaling [6].

In addition to the transcriptional response, hypoxia can also modulate posttranscriptional and posttranslational events. In recent years the importance of mRNA stability and translational control came into focus, and several miRNAs and RNA-binding proteins have been shown to implement the hypoxic gene expression profile [7].

Up to 95% of all human genes are alternatively spliced [8], [9]. Alternative splicing serves as regulatory platform that allows for tissue specific expression and dramatically increases genomic complexity. In addition, aberrant alternative splicing is the cause of several diseases and appears to be involved in cancer progression [10]–[12]. Next to deep sequencing, exon arrays, like the GeneChip Exon Array from Affymetrix, make it possible to assess alternative splicing on a genome-wide scale. In contrast to numerous studies investigating the transcriptional response induced by hypoxia (for examples see [13]–[18]), only two genome-wide analyses of hypoxia-related changes in pre-mRNA splicing have been performed. One identified *lama3* as a hypoxia-related splice variant in head and neck cancers [19]. Another study identified several alternative splice events in endothelial cells using CoCl_2_ as hypoxia mimic and inducer of apoptosis [20].

So far, no genome-wide study investigated changes in splicing events under low oxygen conditions. Therefore, we used the exon array technology to examine hypoxia-related alternative splicing in HUVECs. In combination with RT- and qRT-PCR experiments, we validated alternative splicing for nine genes. None of them had been associated with hypoxia before.

## Results

### Exon array analysis

We performed an Affymetrix GeneChip Human Exon 1.0 ST Array to identify alternative splicing events during hypoxia in HUVECs. In contrast to traditional microarrays, where probes hybridize only to the 3′-end of genes, exon arrays contain probe sets targeting every exon over the entire length of the transcript. One probe set consists of up to four individual probes targeting the same exon. Additionally, larger exons are targeted by multiple probe sets. Therefore, exon arrays allow the simultaneous assessment of whole transcript and single exon levels.

**Figure 1:**
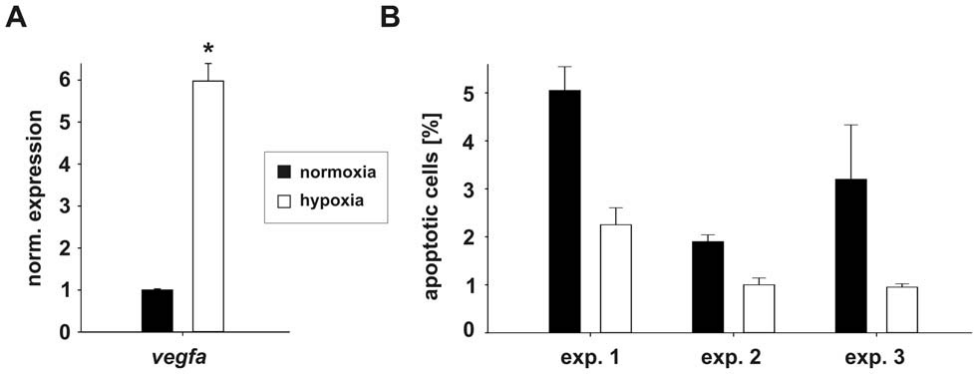
Validation of hypoxic conditions by *vegfa* expression. **A**) Array probes (triplicates) were analyzed for *vegfa* expression using qRT-PCR. *Vegfa* mRNA expression is normalized to the mRNA of the ribosomal Protein RPLP0, which expression does not change under hypoxia. Oxygen starvation (open bar) leads to a 6-fold induction of *vegfa* compared to normoxic conditions (closed bar). * = p-value<0.05 (Student's t-test). **B**) The influence of the hypoxic conditions on apoptosis was assessed. Three independent experiments conducted in duplicates show a decrease of apoptotic cells under hypoxia (open bars) compared to normoxia (closed bars).

Confluent HUVECs were incubated in triplicates for 48 h under normoxic (21% O_2_) or hypoxic (1% O_2_) conditions. Total RNA was isolated and tested for *vegfa* mRNA expression, to confirm the response of the endothelial cells to hypoxia. qRT-PCR analysis showed a 6-fold induction of *vegfa* mRNA levels under hypoxic compared to normoxic conditions (Figure 1A). In addition, the influence of the hypoxic conditions on apoptosis was assessed (Figure 1B). Three independent experiments (conducted in duplicates) show a low percentage of apoptotic cells, with even a slight decrease in apoptosis during hypoxia.

The exon array analysis was conducted on the core probe sets using the Exon Array Analyzer (EAA) Web interface [21]. Analysis was performed with two different algorithms (RMA and Iter-PLIER) for background correction and signal estimation. Only genes/probe sets, which showed at least a 2-fold change in expression and a p-value<0.01 were considered for further analysis.

Using RMA, 296 genes were identified to be differentially expressed. In contrast, Iter-PLIER revealed more than twice the number (650 genes). Comparing the results of the two algorithms, we found that 99% (294) of the genes shown to be differentially expressed using RMA were also identified by Iter-PLIER. Only genes identified by both algorithms were chosen for further analysis.

Of the 294 regulated genes, 86 (29%) are up- and 208 (71%) are downregulated under hypoxic conditions (Tables S1 and S2). GO analysis was performed on these two groups using DAVID Bioinformatics Resources 6.7 (http://david.abcc.ncifcrf.gov/) [22], [23]. Upregulated genes are most highly enriched for genes responsive to hypoxia or related to hexose metabolism (Table 1). Furthermore, several genes for cell migration, angiogenesis, tube development and response to wounding are induced upon hypoxia (Table 1). This relates to the function of HUVECs in blood vessel development. Downregulated genes, however, are almost exclusively related to the cell cycle, DNA replication or DNA repair (Table 2).

**Table 1:** GO analysis of upregulated genes.

GO term	p-value	Gene symbols
0001666∼response to hypoxia	4.0×10^−8^	*adm, aldoc, angptl4, bnip3, cxcr4, egln3, pdgfb, pgf, vegfa, vldlr*
0045429∼positive regulation of nitric oxide biosynthetic process	5.6×10^−3^	*igf2, icam1, insr*
0019318∼hexose metabolic process	7.7×10^−5^	*aldoc, eno2, gbe1, hk2, igf2, pdk1, pfkfb3, pgm1* 1
0015758∼glucose transport	0.011	*igf2, slc2a1, slc2a3*
0032868∼response to insulin stimulus	2.1×10^−3^	*adm, igf2, insr, pparg, vldlr*
0006090∼pyruvate metabolic process	0.022	*eno2, pgm1, slc16a3*
0046942∼carboxylic acid transport	0.045	*pparg, slc16a3, slc6a6, slc7a2*
0042981∼regulation of apoptosis	3.7×10^−3^	*adora2a, angptl4, bnip3, dusp1, igf2, inhba, pde3a, pim1, ptgis, timp3, tnfsf15, vegfa* 2
0008285∼negative regulation of cell proliferation	0.013	*adm, adora2a, mxi1, nox4, pparg, tgfb1i1, tnfsf15*
0030335∼positive regulation of cell migration	1.2×10^−4^	*icam1, igf2, insr, pdgfb, vegfa, vegfc*
0001525∼angiogenesis	1.2×10^−3^	*angptl4, cxcr4, cyr61, pgf, vegfa, vegfc*
0035295∼tube development	6.6×10^−3^	*cxcr4, cyr61, lox, pgf, spry1, vegfa*
0009611∼response to wounding	7.7×10^−3^	*adm, adora2a, cxcr4, fbln5, igf2, lox, nox4, pdgfb, timp3*
0008015∼blood circulation	0.018	*adm, adora2a, npr3, pparg, vegfa*
0030198∼extracellular matrix organization	0.019	*cyr61, fbln5, lox, p4ha1*
0007568∼aging	0.022	*adm, aldoc, nox4, timp3*

1 Underlined genes match to the GO term: 0006096∼glycolysis (p-value = 2.1×10^−3^).

2 Underlined genes match to the GO term: 0043066∼negative regulation of apoptosis (p-value = 0.012).

**Table 2:** GO analysis of downregulated genes.

GO term	p-value	Gene symbols
0007049∼cell cycle	2.9×10^−42^	*anln, aspm, aurka, aurkb, brca1, bub1, bub1b, ccna2, ccnb1, ccnb2, ccnd1, ccne2, cdc2, cdc20, cdc45l, cdc6, cdca2, cdca8, cdkn3, cenpe, cenpf, cep55, ckap2, cks2, clspn, dhcr24, dlgap5, exo1, fam83d, fancd2, fbxo5, foxm1, gmnn, hells, hjurp, kif11, kif15, kif18a, kif20b, kif23, kif2c, map2k6, mcm6, mki67, ncapd3, ncapg, ncaph, ndc80, nuf2, nusap1, pbk, plk1, prc1, pttg1, racgap1, rad51, sesn3, sgol1, sgol2, ska1, ska3, spag5, spc25, tacc3, tpx2, trip13, ttk, txnip, uhrf1, zwilch, zwint*
0006260∼DNA replication	2.8×10^−14^	*brca1, ccne2, cdc45l, cdc6, cenpf, clspn, dtl, fen1, gins1, gins2, gins3, mcm10, mcm4, mcm6, pcna, pole2, rad51, rfc3, rrm2, tk1, top2a, tyms*
0006281∼DNA repair	6.2×10^−7^	*brca1, brip1, clspn, exo1, fancd2, fen1, neil3, pcna, pole2, pttg1, rad51, rad51ap1, rfc3, top2a, trip13, tyms, uhrf1*
0031497∼chromatin assembly	1.5×10^−6^	*asf1b, hells, hist1h1a, hist1h1b, hist1h2al, hist1h2bf, hist1h2bm, hist1h3a, hist1h3b, hist1h3c, hist1h3h, hist1h3j, hist2h2ab, hjurp*
0006979∼response to oxidative stress	0.018	*dhcr24, hmox1, nqo1, pla2g4a, stat1, txnip, ucp2* 1
0042981∼regulation of apoptosis	0.048	*brca1, cdc2, dhcr24, hells, hmox1, hspa1a, map2k6, nme1, nqo1, pla2g4a, serpinb2, sgk3, sort1, stat1, tnfsf18, top2a, txnip* 2
0048015∼phosphoinositide-mediated signaling	1.5×10^−7^	*aurka, bub1b, cks2, fen1, ndc80, pcna, pik3c2b, spag5, top2a, tyms, zwint*

1 Underlined genes match to the GO term: 0000302∼response to reactive oxygen species (p-value = 0.015).

2 Underlined genes match to the GO term: 0043066∼negative regulation of apoptosis (p-value = 0.036).

When comparing our gene expression changes with a microarray performed by Scheurer *et al.* [16], where HUVECs were kept under similar hypoxic conditions (48 h, 2% O_2_), the results agree remarkably, despite the fact, that different array platforms have been used. 246 genes out of the 294 identified genes are also detected by the microarray platform. From these 57% and 63%, respectively, of the up- and downregulated genes are identified in both studies with a p-value<0.01. In comparison using the same selection criteria as used in our study (>2-fold change, p-value<0.01, predicted by both RMA and Iter-PLIER) only 3% of the upregulated and 25% of the downregulated genes are found in the study by Hang *et al.* [20], who used the same exon array platform to study alternative splicing in HUVECs using CoCl_2_ as hypoxia mimic and inducer of apoptosis. A similarity is that in all three studies considerably more genes are downregulated (between 2.4- and 4.5-fold more).

### Hypoxia-induced alternative splicing

**Figure 2:**
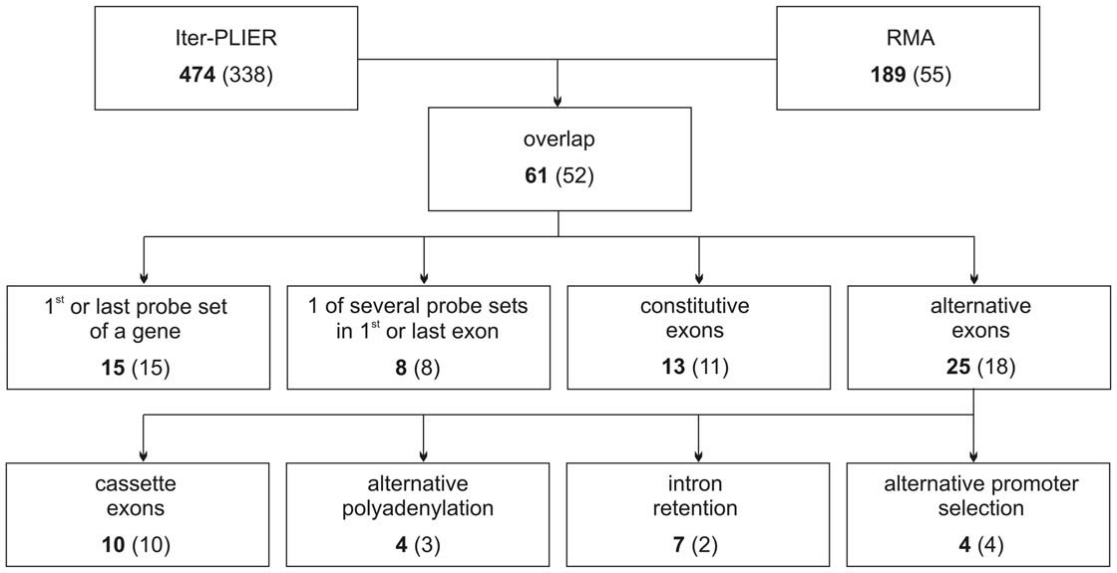
Analysis of hypoxia induced alternative splicing. Differentially expressed probe sets are given in bold. The number of the corresponding genes is given in brackets.

Analysis of the expression of individual probe sets with RMA showed 189 probe sets, whereas Iter-PLIER showed 474, with a change of expression of >2-fold and a p-value<0.01 (Figure 2). In contrast to the results on transcript level, where nearly all genes found by RMA are also identified by Iter-PLIER, the overlap on probe set level is only 32%. The calculation of the differential expression of individual probe sets depends on the normalized probe set as well as the normalized gene level. Therefore, different background correction strategies as implemented in RMA and Iter-PLIER affect the expression signals at both levels, ultimately resulting in different predictions. Still we think that combination of both algorithms is beneficial, due to the fact, that both have different strength and limitations in the detection of alternative splicing events [24]. The 61 differentially expressed probe sets identified by both algorithms are distributed over 52 genes (Table S3) and cluster in four groups (Figure 2):

15 probe sets are the very first or very last probe set on the respective mRNA. Here, differential expression would lead to shorter or longer untranslated regions (UTRs) potentially modulating gene expression. We tested six of these probe sets for differential expression by comparing their expression under normoxic and hypoxic conditions to the rest of the mRNA. Primer pairs were designed amplifying either the extreme 5′ (*adssl1*, *dnmt1*, *mme, ucp2*) or 3′ (*sdc2*, *smarca1*) ends (the region indicated by the putative differentially expressed probe set) or a section of the open reading frame (ORF). All primer pairs showed a clear signal, but none of the UTR probes showed an expression different to the corresponding ORF (see for example *ucp2*, Figure S1).

In eight cases only one of several probe sets corresponding to the first or last exon of a mRNA shows differential expression, while the other probe sets remain at a constant level. We tested two (*vegfa*, *adamts1*) of these exons for differential expression, but they behave like the remainder of the respective mRNA (induction for *vegfa*, reduction for *adamts1*). Therefore we speculate that most of the probe sets from these two groups are false positives.

13 changed probe sets correspond to exons annotated as constitutive. They were not analyzed further, albeit it is possible that they are indeed alternatively spliced. In seven cases, skipping of the constitutive exon would target the mRNA to nonsense mediated decay and consequently not lead to protein expression. The other six cases could produce an alternative protein isoform (Table S3).

25 of the 61 probe sets correspond to known exon skipping, intron retention, alternative promoter or polyadenylation events (Figure 2). We chose 19 of these for further validation.

### Alternatively spliced cassette exons

**Figure 3:**
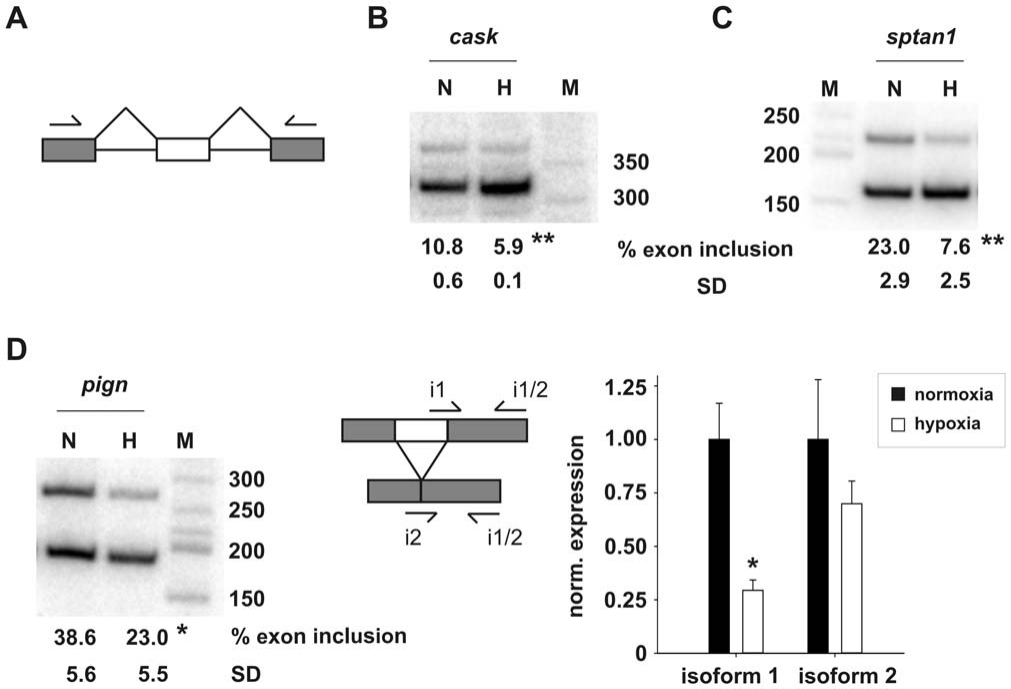
Hypoxia-dependent alternative exon inclusion. **A**) Scheme of alternative splicing of cassette exons. Introns are represented as black lines; constitutive exons as grey boxes and the cassette exon as white box. Primers are indicated as arrows and span the alternatively spliced exon. Cassette exons in *cask* (**B**), *sptan1* (**C**) and *pign* (**D**) are more often skipped under hypoxic then under normoxic conditions. Radioactive RT-PCR: N = normoxia, H = hypoxia, M = DNA size ladder. The precentage of exon inclusion is given below. SD = standard deviation. qRT-PCR: closed bars = normoxia, open bars = hypoxia. Two isoform specific forward primer (i1 and i2) spanning the exon/exon borders and one common reverse primer (i1/2) were used. Expression is normalized to *rplp0*. Results from n = 3 experiments are shown. * = p-value<0.05, ** = p-value<0.01 (Student's t-test).

Cassette exons account for about 40% of the alternative splicing events in higher eukaryotes [25]. We tested six of the ten predicted alternatively spliced cassette exons for differential expression under normoxic and hypoxic conditions. We validated alternative splicing for *cask*, *sptan1* and *pign* by radioactive RT-PCR (Figure 3) and for *pign* also by isoform-specific qRT-PCR (Figure 3D). In all three genes the cassette exons are included at a lower frequency upon hypoxia treatment. The predicted alternative splicing event could not be validated for *acot7*, *rfx3* and *stil* (data not shown). Here, either only one isoform was detected or there was no change in the ratio of the two isoforms comparing expression at normoxia and hypoxia.

CASK (calcium/calmodulin-dependent serine protein kinase) is a membrane-associated guanylate kinase located at neuronal synapses and cell-cell-junctions. Mutations result in X-linked mental retardation [26] and the knockout of *cask* in mice is neonatal lethal [27]. CASK is a multidomain protein coding N-terminal for a CaM kinase II domain and C-terminal for PDZ, SH3 and guanylate kinase-like domains. CASK binds the proangiogenic factor SDC2 (syndecan 2) [28], [29] as well as the cytoskeletal adaptor protein EPB41 (protein 4.1) [28] and thereby links components of the extracellular matrix to the intracellular cytoskeleton. It is also involved in the regulation of cell growth [30] and transcription [31]. The two detected isoforms of CASK (Figure 3B) differ in 23 amino acids (aa) in the linker region between the PDZ and SH3 domains (Figure S2A). In this case, alternative splicing modulates domain spacing, which may influence the ability of both domains to engage in protein binding simultaneously. In addition, the skipped linker region harbors a putative PEST-Element, which might influence proteasome-dependent degradation [32]. As a result, alternative splicing would directly impact on protein stability.

S*ptan1* (alpha-II spectrin or alpha-fodrin) is specifically expressed in non-erythrocytic cells. Spectrins are essential components of the cytoskeleton, composed of alpha- and beta-spectrins, which form heterotetramers and directly bind to filamentous actin. Spectrins associate with EPB41, WAS (Wiskott-Aldrich syndrome protein, also WASP) and junction proteins like TJP1 (tight junction protein 1, also ZO-1), CTNNA1 (alpha-catenin) and GJA1 (gap-junction protein alpha 1, also connexin-43). Apart from several spectrin repeats, which allow for tetramerization with the beta-subunit, *sptan1* codes for two EF hand motives and an SH3 domain. The two detected isoforms (Figure 3C) differ in a 20 aa insertion C-terminal of the SH3 domain (Figure S2B). The longer isoform, which is downregulated under hypoxic conditions, has been shown to associate with GJA1 and to be required for its localization to gap junctions [33]. The binding depends specifically on the 20 aa insertion and may be sensitive for phosphorylation by MAPK8 (JNK).

PIGN is involved in the glycosylphosphatidylinositol (GPI)-anchor biosynthesis. It transfers phosphoethanolamine to the first mannose of the GPI-anchor. The GPI-anchor is a glycolipid attached to the C-terminus of target proteins in the endoplasmatic reticulum. After secretion GPI-anchored proteins stay attached to the membrane and protrude into the extracellular space. Anchored proteins include the proangiogenic factors CDH13 (T-cadherin) [34] and EPHA1 (ephrin A1) [35]. Alternative splicing of *pign* does not result in different protein isoforms, but in two 5′UTRs, which differ in 77 nucleotides (nt) in length (Figure 3D). Hypoxia downregulates *pign* expression; especially the longer 5′UTR isoform. Different 5′UTRs can impact on the translational efficiency of the mRNA, due to secondary structures, protein binding sites, internal ribosome entry sites or upstream open reading frames (uORFs). Therefore, the alternative splicing of the 5′UTR of *pign* might influence protein expression under hypoxia.

### Alternative polyadenylation

**Figure 4:**
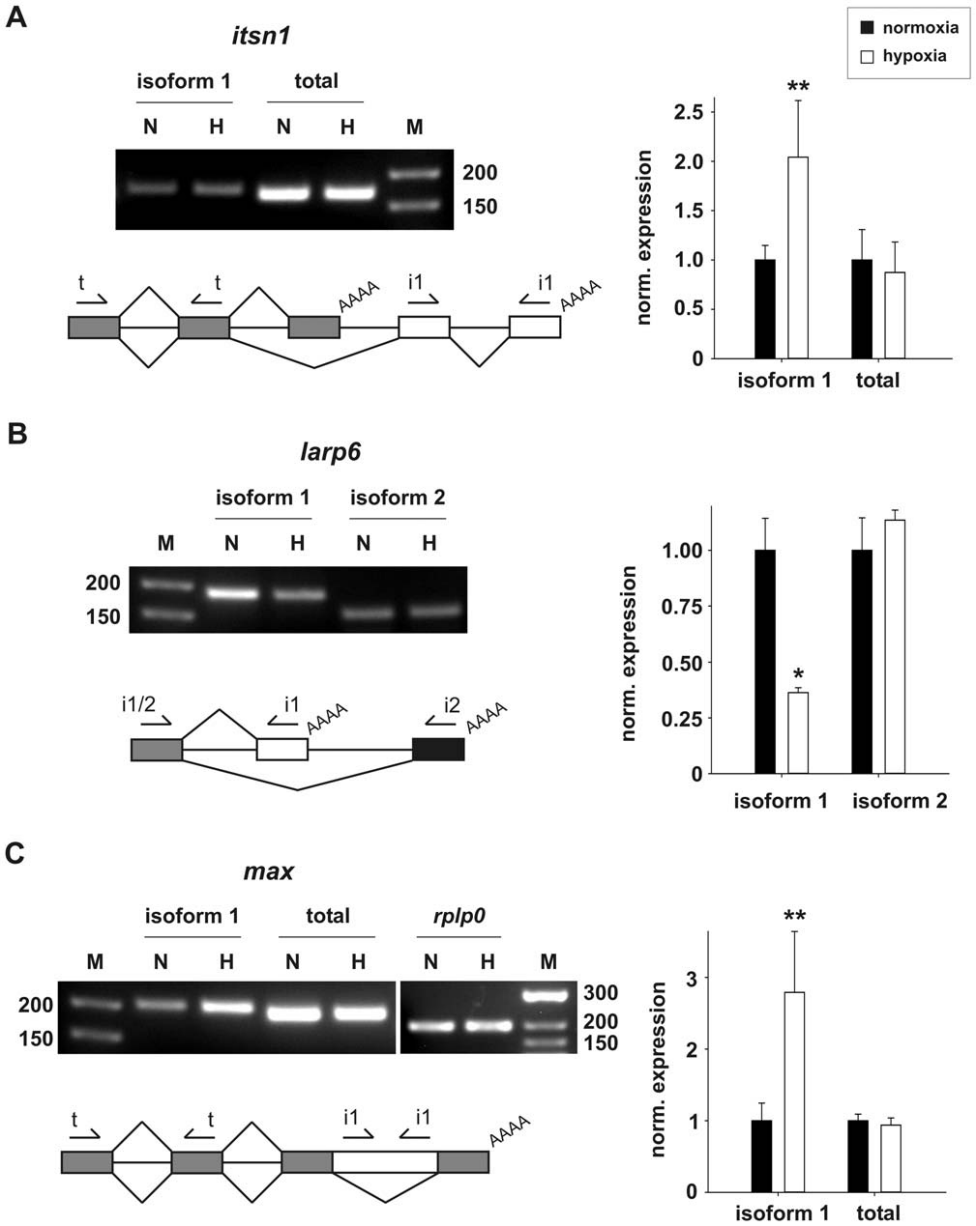
Hypoxia-dependent alternative C-termini. RT- and qRT-PCR analyses show changed expression of protein isoforms for *itsn1* (**A**), *larp6* (**B**) and *max* (**C**). Alternative splicing and polyadenylation lead to the expression of isoforms with different C-termini in *istn1* (**A**) and *larp6* (**B**). In the case of *max* (**C**) due to intron retention an alternative stop codon is introduced, also leading to a different C-terminus. Schemes of alternative splicing events: Introns are represented as black lines; exons as boxes; primer pairs (arrows) used to amplify different isoforms (i1, i2) or total amounts of the mRNAs (t) are indicated. White exons indicate isoform 1, which expression is changed under hypoxic conditions. Grey exons are common to both isoforms and primers spanning such regions were used to visualize changes in the total amount of the corresponding mRNA. In the case of *larp6* only the first exon is common to both isoforms, therefore isoform 2 (black exon) was visualized instead of the total mRNA amount, using one common forward- and two isoform-specific reverse-primers. The primer pair recognizing the retained intron in the *max* mRNA could yield the same product with genomic DNA. To ensure sample purity and exclude falsification of the results by contamination with genomic DNA, we performed the same RT- (Figure S5) and qRT-PCR experiments with not reverse transcribed RNA (data not shown). These experiments yielded no product at all confirming the observed increase is due to intron retention. RT-PCR: N = normoxia, H = hypoxia, M = DNA size ladder. qRT-PCR: closed bars = normoxia, open bars = hypoxia. Expression is normalized to *rplp0*. Results from n = 2 (RT-PCR) and n = 3 (qRT-PCR *larp6*) or n = 6 (qRT-PCR *itsn1* and *max*) experiments are shown. * = p-value<0.05, ** = p-value<0.01 (Student's t-test).

More than 50% of all human genes exhibit multiple poly(A) sites [36]. Alternative polyadenylation (APA) can affect 3′UTR length, influencing e.g. mRNA stability by changing miRNA and protein binding sites, or changing the protein coding capacity of an mRNA. APA is regulated during proliferation and differentiation and aberrant APA can be the causative agent of cancer [37]. Often APA is associated with alternative splicing. We tested two of the three predicted APA events (Table S3) and validated both (see *itsn1* in Figure 4A and S3A; *larp6* in 4B and S3B). In both cases alternative splicing and APA lead to an altered C-terminus of the encoded protein.

ITSN1 (intersectin 1) is an adaptor protein involved in endo- and exocytosis, signal transduction and cytoskeleton remodeling. *Itsn1* codes for a long (ITSN1-L) and a short protein isoform (ITSN1-S). Hypoxia specifically leads to the upregulation of the long isoform ITSN1-L (Figure 4A), but not of ITSN1-S. ITSN1 knockout mice, either a complete knockout or only knockout of ITSN1-L, are viable but show defects in vesicle trafficking and alterations in the level of NGF (nerve growth factor) [38]. ITSN1-S consists of two EH1 domains, a coiled-coil region and five SH3 domains. The by ten exons longer isoform ITSN1-L contains three additional domains: DH (DBL homology domain), PH and C2 and shows GEF (guanine nucleotide exchange factor) activity towards the proangiogenic factor CDC42, bringing it into the active GTP-bound form [39].

LARP6 (La-related protein 6 or acheron) is structurally related to La/SSB (Lupus erythematosus and Sjogren Syndrome antigen). It codes for three La motives and one RRM domain, which both are important for RNA-binding. It regulates the translation of collagen mRNAs by binding to a conserved stem-loop in the 5′UTR [40]. LARP6 was shown to regulate cell adhesion and motility in myoblasts [41], as well as cell proliferation and apoptosis in endothelial cells [42]. *Larp6* codes for two protein isoforms: one full-length (isoform 2 in Figure 4B) and one short isoform (isoform 1), which is downregulated under hypoxic conditions (Figure 4B). Isoform 1 is highly expressed under normoxic conditions, but does not code for any known protein domain. It consists of only two exons: it shares exon 1 (66 aa) with the full-length protein and contains a unique exon 2, which codes for additional 27 aa. Exon 2 does not seem to be evolutionary conserved, not even among primates.

### Intron retention

Intron retention is the rarest alternative splicing event in humans [25]. We tested both of the introns predicted to be differentially expressed (Table S3) and validated alternative splicing for *max* (Figure 4C and S4). The retained intron in the 3′UTR of *zwint* was expressed at a similar level as the remaining mRNA, but did not show an increase under hypoxic conditions. In contrast, it showed the same reduction as the complete mRNA (Table S2).

*Max* (myc associated factor X) encodes a transcription factor of the basic helix-loop-helix leucine zipper family. MAX can either form homodimers or heterodimers with other family members, like MXD1 (also MAD), MXI1, MNT and MYC. The different types of dimers compete for a common binding site (E box) in promoter regions. MAX-MYC dimers transcriptionally activate the target genes, leading to cell proliferation, but also sensitize cells to apoptosis [43]. In contrast, heterodimers with other factors, like the upregulated MXI1 (Table S1) act as transcriptional repressors, therefore (at least partially) counteracting MYC activity [43]. In endothelial cells overexpression of MAX antagonizes serum-induced apoptosis [44]. Intron retention between exons 4 and 5 leads to an altered C-terminus of the protein (Figure 4C). The hypoxia-induced isoform (isoform 1) shares the first 98 aa with the full-length protein (160 aa). It codes almost for the complete DNA-binding domain, but contains a unique C-terminus (36 aa). How the altered C-terminus impacts on protein function is unknown. A different C-terminal isoform, where only part of the intron is retained, and which also contains the first 98 aa, is still capable of MYC-binding [45]. However, in contrast to the full-length protein, it harbors no functional nuclear localization signal, but relies on nuclear translocation by binding to MYC. In addition, instead of repressing the transforming activity of MYC, like full-length MAX, it enhances it.

### Alternative promoter selection

**Figure 5:**
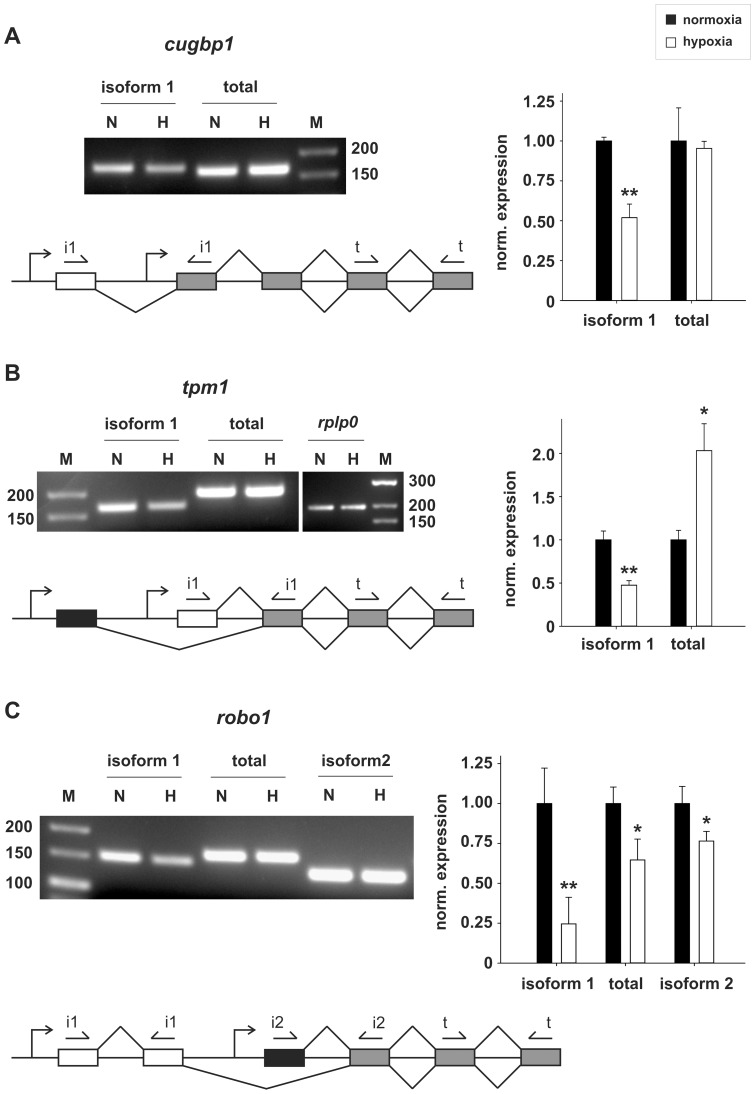
Hypoxia-dependent alternative N-termini. RT- and qRT-PCR analyses show alternative promoter usage for *cugbp1* (**A**), *tpm1* (**B**) and *robo1* (**C**) leading to protein isoforms with different N-termini. Schemes of alternative splicing events: Introns are represented as black lines; exons as boxes; primer pairs (arrows) used to amplify different isoforms (i1, i2) or total amounts of the mRNAs (t) are indicated. White exons are exclusive for isoform 1, which expression is changed under hypoxic conditions. Grey exons are common to both isoforms and primers spanning such regions were used to visualize changes in the total amount of the corresponding mRNA. Black exons are exclusive for isoform 2, which is transcribed by an alternative promoter. RT-PCR: N = normoxia, H = hypoxia, M = DNA size ladder. qRT-PCR: closed bars = normoxia, open bars = hypoxia. Expression is normalized to *rplp0*. Results from n = 2 (RT-PCR) and n = 3 (qRT-PCR) experiments are shown. * = p-value<0.05, ** = p-value<0.01 (Student's t-test).

30–50% of all human genes are transcribed from more than one promoter [46]. Alternative promoter selection (APS) leads to the expression of mRNA isoforms with different 5′UTRs, implicated in translation efficiency, or ORFs, which differ in their N-termini. Aberrant use of alternative promoters has been studied in several disease-related genes and is linked to cancer progression [46]. We tested three (*cugbp1*, *tpm1*, *robo1*) of the four APS events triggered through hypoxia and validated all of them (Figure 5).

*Cugbp1* (CUG-repeat binding protein 1 or CELF1) encodes a RNA-binding protein comprised of two N-terminal and one C-terminal RRM domains, separated by a serine-rich region. CUGBP1 is implicated in alternative splicing, mRNA decay and translation. A recent study identified over 600 targets of CUGBP1 by co-immunoprecipitation [47]. Many contain so-called GRE (GU-rich element) sequences in their 3′UTR, which are bound by CUGBP1 and promote rapid degradation of the mRNA. Cardiac-specific overexpression in adult mice results in premature lethality and cardiomyocyte pathology [48]. Hypoxia leads to the downregulation of an N-terminally prolonged protein isoform (isoform 1, Figure 5A). It is unknown how these additional 27 aa impact on protein function. However, evolutionary conservation and differential expression from the different promoters during mouse myogenesis [49] hints to a distinct functionality of the hypoxia regulated isoform.

TPM1 (alpha-tropomyosin) is an actin-binding protein, involved in the contractile system of muscles and the cytoskeleton of non-muscle cells. It consists of two alpha-helices assembled into dimeric coiled-coils. *Tpm1* (and also the other three tropomyosin genes) is transcribed from two independent promoters, leading to the expression of high (HMW) and low (LMW) molecular weight isoforms. In addition, alternative splicing generates more than 40 tropomyosin isoforms in non-muscle cells. The various tropomyosin isoforms have a different intracellular localization, leading to functionally distinct actin filament populations [50]. Hypoxia specifically downregulates the LMW isoforms of *tpm1* (isoform 1, Figure 5B), but upregulates the total amount of alpha-tropomyosin. The increase in *tpm1* expression was correctly predicted by both Iter-PLIER (2.01-fold, p-value<0.01) and RMA (1.67-fold, p-value<0.01), but is not included in Table S1, due to the less then 2-fold induction predicted by RMA. This increase results in a shift towards HMW isoforms, which have been shown to possess tumor suppressor functions and are downregulated in cancer cells [50]. LMW isoforms probably function in cell-cell adhesion and exocytosis [50]. In addition, tropomyosins regulate actin filament dynamics, impacting on cell migration by interfering with Arp2/3 driven actin branching [51].

*Robo1* (roundabout 1) codes for a membrane receptor mediating SLIT-induced repulsion on axon guidance, which plays an important role in angiogenesis [52]. The extracellular part of ROBO1 contains five immunoglobulin-like and three fibronectin type III domains. The protein further codes for one transmembrane region, as well as four conserved motives and a serine-rich region in the cytoplasmic part. Hypoxia downregulates *robo1* expression, especially the N-terminally longer isoform (isoform 1, Figure 5C). A slight downregulation to around 60% (p-value<0.01) of the total amount of robo1 expression was correctly predicted by both Iter-PLIER and RMA. The SLIT ligands bind to the first two Ig domains in the N-terminal part of the protein [53]. Therefore, the N-terminal extension might impact on ligand affinity. Moreover, the reduction of isoform 1 may interfere with the known homo- and heterodimerization of ROBO receptors. Differential expression of two N-terminally distinct isoforms has also been shown for ROBO2 and 3 [54], [55]. In zebrafish the ROBO3 isoforms have distinct functions in axon pathfinding and dorsoventral cell fate specification [54]. For the human ROBO3 isoforms differential SLIT binding has been shown *in vitro* [56]. The function of the two ROBO1 isoforms is unknown, but evolutionary conservation and differential expression during mouse embryogenesis [57], [58] hint at distinct functionalities.

Taken together, we show alternative splicing of nine genes upon hypoxic treatment in endothelial cells; resulting in eight different protein isoforms and one alternative 5′UTR. Altogether 27 probe sets were tested for differential expression and 15 of them confirmed, corresponding to a validation rate of 56%. Considering only probe sets related to known alternative exons (19 probe sets) the validation rate amounts to 80%, due to the fact that all 15 validated probe sets belong to this group.

## Discussion

We analyzed gene expression and splicing changes in endothelial cells subjected to oxygen starvation (1%) for 48 h and showed that hypoxia significantly changed alternative splicing decisions. Whereas one previous study addressed the effect of hypoxia-induced apoptosis on alternative splicing in endothelial cells [20], this is to our knowledge the first study addressing the regulation of splicing under physiological adaptation. Consistently, several genes for which we showed alternative splicing are involved in the regulation of cytoskeletal remodeling and migration.

Analyzing the functions of the genes validated to undergo alternative splicing, we find several overlaps, like cellular localization and involvement in cell motility. Two of the alternatively spliced genes are essential parts of the cytoskeleton (*sptan1* and *tpm1*), which has to be remodeled to allow for cell migration and angiogenesis. The downregulated splice isoform of *sptan1* has been shown to be important for the localization of GJA1 (connexin 43) to gap junctions in cardiomyocytes [33]. Previous studies have shown that hypoxia reduces GJA1 expression, phosphorylation and localization [59], [60]. Downregulation of GJA1 increases healing in a fibroblast wound-healing model by accelerating cell migration and proliferation [61]. In addition, Danesh-Meyer *et al.* [62] demonstrated that endothelial cell death following hypoxia can be mediated by GJA1 hemichannel opening. We therefore speculate, that alternative splicing of *sptan1* positively contributes to cell survival and/or angiogenesis by the reduction of gap junctions.

CDC42 is a small GTPase regulating actin polymerization by signaling via WAS and WASL (also NWASP) to the Arp2/3 complex [63], which in turn is also regulated by the alternatively spliced TPM1 [51]. CDC42 induces filopodia formation and is specifically activated by the splice isoform of ITSN1 (ITSN1-L) [39], which is upregulated by hypoxia. Therefore, alternative splicing of ITSN1 likely leads to activation of the proangiogenic factor CDC42.

CDC42 is also important for SDC2 signaling [64]. SDC2 is the main heparan sulfate proteoglycan expressed on endothelial cells and important for several steps of neovascularization [65]. CASK is a multi-adaptor protein connecting SDC2 to the cytoskeleton and has been shown to stabilize dendritic spines, developed from SDC2-induced filopodia, in neurons [66]. CASK also binds to LARP6 and seems to be connected to its positive effect on HUVEC cell proliferation [42], [67]. In addition, SDC2 has been shown to enhance ligand binding to ROBO1 [68]. ROBO1 heterodimerizes with the endothelial specific ROBO4 and positively regulates angiogenesis under normoxic conditions [69]. ROBO4 in turn has been shown to bind to WAS and WASL in HUVECs [69]. The effects of the alternative splicing events in *cask*, *larp6* and *robo1* remain unclear, however, we can speculate that they might be involved in angiogenesis through activation of CDC42.

When comparing our study with previously reported hypoxia-related alternative splicing events, we find no overlap: *lama3* was identified in head and neck cancers [19], *hif3a* in mouse heart [70], *bnip3* in cardiac myocytes [71], *cyr61* in several tumor cell lines [72], *psen2* in neuroblastoma cells [73] and *pfkfb3* when comparing different hypoxic mouse tissues [74]. This underlines the importance and exclusiveness of tissue specific alternative splicing events. But even when using the same cell line different hypoxic conditions can trigger different splicing decisions: exon array analysis following hypoxia-induced apoptosis in HUVECs identified 15 alternatively spliced genes [20], none of these were identified in our analysis. In addition, we previously identified a change in the ratio of short versus long *flt1* mRNA isoforms after hypoxia treatment [75]. Here HUVECs were incubated under acute (0.1% O_2_) hypoxia, compared to the milder more physiologic condition used in this study (1% O_2_), which does not trigger alternative splicing of *flt1* (data not shown). It seems that more severe hypoxia, which is accompanied by apoptosis results in different splicing events in endothelial cells.

Using very stringent selection conditions (>2-fold change, p-value<0.01 and identification by two algorithms) only few alternative splicing events (61 probe sets) were predicted in this study. Identification of further splicing events could be achieved e.g. by increase of the p-value to <0.05, which would result in around 3-fold more probe sets predicted to change expression. In addition, we analyzed the highly curated core probe sets, but extension of the analysis to all probe sets on the array will show further alternative splicing events. Alternatively, RNA-seq could be used for a complementary genome-wide study. RNA-seq has been shown to be more sensitive in the detection of low abundant exons [76]. In addition, direct comparison of the exon array technology with RNA-seq showed only a small overlap of the predicted alternative splicing events [76]. Therefore, a study using RNA-seq might reveal further events during hypoxia.

In addition to provide novel insights into the changes of alternative splicing in response to hypoxia, the results of the gene expression analysis are consistent with previous studies. Thus, under hypoxic conditions nearly 300 genes change their expression at least 2-fold. More than 200 of these genes are downregulated and GO analysis revealed that most of them are implicated in cell cycle and DNA replication. It has already been demonstrated that hypoxia leads to G1 cell cycle arrest in different cell types, by activation of cyclin-dependent kinase inhibitors and downregulation of cyclins [77], [78]. A microarray-based study analyzed hypoxia-induced gene expression changes in HUVECs at different time points [16]. Also here, prolonged hypoxia (48 h, 2% O_2_) led to a pronounced decrease of cell cycle-related genes, several of which were also identified in the present study. In addition, it is known, that hypoxia downregulates DNA repair mechanisms [79], like mismatch repair, base excision repair, homologous recombination and DNA-damage checkpoints of the cell cycle (see Table 2 for examples). Upregulated genes are most highly enriched in known hypoxia-related genes (Table 1), but also in genes involved in hexose metabolism and angiogenesis, both well-established features of hypoxia. Thus, the lists of up- and downregulated genes recapitulate known responses to hypoxia.

## Methods

### Cell culture

Pooled HUVECs (Lonza) were grown in endothelial cell basal medium (EBM), supplemented with EGM SingleQuots (both Lonza) and 10% FCS (Gibco) in T75 flasks (Greiner). At the third passage 1×10^6^ cells were seeded into 6 cm dishes (Greiner), after 18–24 h attachment supplied with fresh medium and incubated for additional 48 h at normoxia or hypoxia (1% O_2_ in a Binder CO_2_/O_2_ incubator).

### RNA isolation

Total RNA was isolated using the miRNeasy Mini kit, including the optional on-column DNA digestion with the RNase-Free DNase Set (both Qiagen). After isolation 1 µg RNA was quality checked on a 1% agarose gel.

### Exon Array

Exon arrays were performed with three independent replicate samples for each experimental condition by ATLAS Biolabs (Berlin). Data analysis was performed on the raw CEL files using the Exon Array Analyzer (EAA) Web interface (http://eaa.mpi-bn.mpg.de/) [21] with the following settings: Species – Human; Gene and Exon set – both core; Gene and Exon level normalization – both either Iter-PLIER or RMA. The EAA uses the Affymetrix Power Tools (APT) for preprocessing. Here either RMA or Iter-PLIER were applied for normalization, background correction and summarization to estimate exon and gene intensities. The APT can also calculate the Detection Above Background (DABG) p-values for each probe set. Before alternative splicing is analyzed five filters are applied to reduce false positives: (i) probe sets that are not expressed in one group are removed. Probe sets are considered as “not expressed”, if their DABG p-value is >0.05 in >50% of the samples in one treatment group; (ii) transcript clusters that are not expressed in both groups are removed. Transcript clusters are considered “not expressed”, if >50% of their probe sets are undetected in >50% of samples in one treatment group; (iii) probe sets for high potential for cross-hybridization are discarded; (iv) transcript clusters with >10-fold difference in gene expression are removed and (v) probe sets with very large gene-level normalized intensity (>5) are removed. Then, the Splice index (SI) is calculated for individual probe sets, to analyze alternative splicing: SI = log_2_ (NI treatment group 1/NI treatment group 2), with NI (gene-level normalized intensity) = probe set intensity/transcript cluster intensity. Only transcript clusters/probe sets changing>2-fold, with a p-value of <0.01 and predicted with both algorithms were chosen for further analysis. The exon array data are available at the GEO database (accession number GSE36837).

### RT-PCR for mRNA isoform validation

For cDNA preparation 1 µg total RNA was reverse transcribed using random hexamer primers (Fermentas) or oligo(dT) (T_12_, Sigma-Aldrich) and MuLV reverse transcriptase (Applied Biosystems) in the supplied buffer system (10 min at 25°C, 15 min at 42°C, 5 min at 99°C). After synthesis the reaction was diluted to a final volume of 200 µl. For non-radioactive RT-PCR 5 µl cDNA were used in a 30 µl PCR reaction with Taq polymerase (New England Biolabs) in the supplied buffer system (95°C 30 sec, 55°C 30 sec, 72°C 30 sec, 32–38 cycles) and the products analyzed on a 3% agarose gel. Every isoform was confirmed in the samples used for the exon array and one RNA sample from an independent experiment (n = 2). For radioactive RT-PCR the forward primer was labeled using γ-^32^P-ATP (Hartmann Analytic) and T4 Polynucleotide-Kinase (Roche) in the supplied buffer system. Prior to use primers were purified using MicroSpin G-25 columns (GE Healthcare) and diluted 1∶10 with unlabeled primer. The same PCR conditions as for non-radioactive RT-PCR were used (30 cycles) and the products analyzed on a 6% polyacrylamid gel (n = 3). Exon inclusion was quantified using ImageQuant and a Typhoon Phosphoimager (GE Healthcare). Primer sequences are listed in Table S4. For validation all bands were gel purified and subcloned for sequencing using the CloneJET PCR Cloning Kit (Fermentas).

### qRT-PCR quantification

qRT-PCR was performed on a StepOnePlus Real-Time PCR System using the Fast SYBR Green Master Mix (both Applied Biosystems). Primers are listed in Table S4. As internal control the mRNA of *rplp0* was used. For qRT-PCR quantification of mRNA isoforms three to six RNA samples were prepared from independent experiments other than for the exon array and RT-PCR (n = 3 or 6). Results are given as mean values ± SD.

### Analysis of apotosis

Cells were grown in duplicates under normoxic and hypoxic conditions (n = 3). Apoptosis was assessed using the FITC BrdU Flow Kit (BD Biosciences) according to the manufacturer's protocol. Fixed and stained cells were analyzed on a fluorescence activated cell sorter (FACS Canto II, BD Biosciences). Results are given as mean values ± SD.

## Supporting Information

Figure S1 **RT-PCR analysis of** ***ucp2*** **expression.**
(TIF)

Figure S2 **Protein domains of CASK and SPTAN1.**
(TIF)

Figure S3 **qRT-PCR analysis of APA in** ***itsn1*** **and** ***larp6*** **.**
(TIF)

Figure S4 **RT-PCR analysis of the intron retention in the** ***max*** **mRNA.**
(TIF)

Figure S5 **Minus RT control for the intron retention in the** ***max*** **mRNA.**
(TIF)

Table S1 **List of upregulated genes.**
(PDF)

Table S2 **List of downregulated genes.**
(PDF)

Table S3 **List of alternatively expressed probe sets.**
(PDF)

Table S4 **Primer sequences used in this study.**
(PDF)
