# Supplementary figures and images for: Hypoxia-Induced Alternative Splicing in Endothelial Cells
Source: PLoS One. 2012 Aug 2;7(8):e42697. doi: 10.1371/journal.pone.0042697 (PMC3411717; doi:10.1371/journal.pone.0042697)

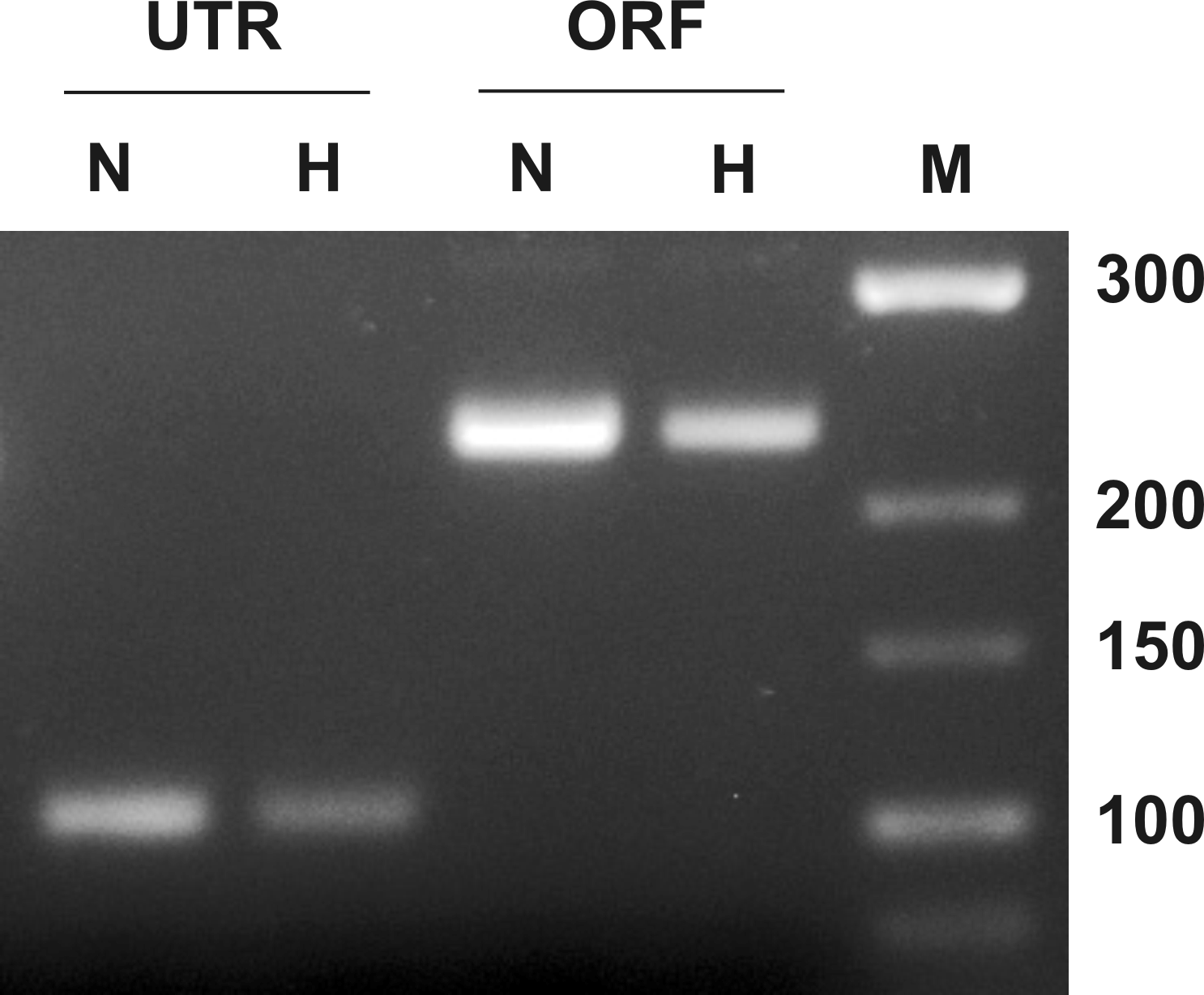

Supplement: Figure S1 — RT-PCR analysis of ucp2 expression. (TIF) [file pone.0042697.s001.tif]

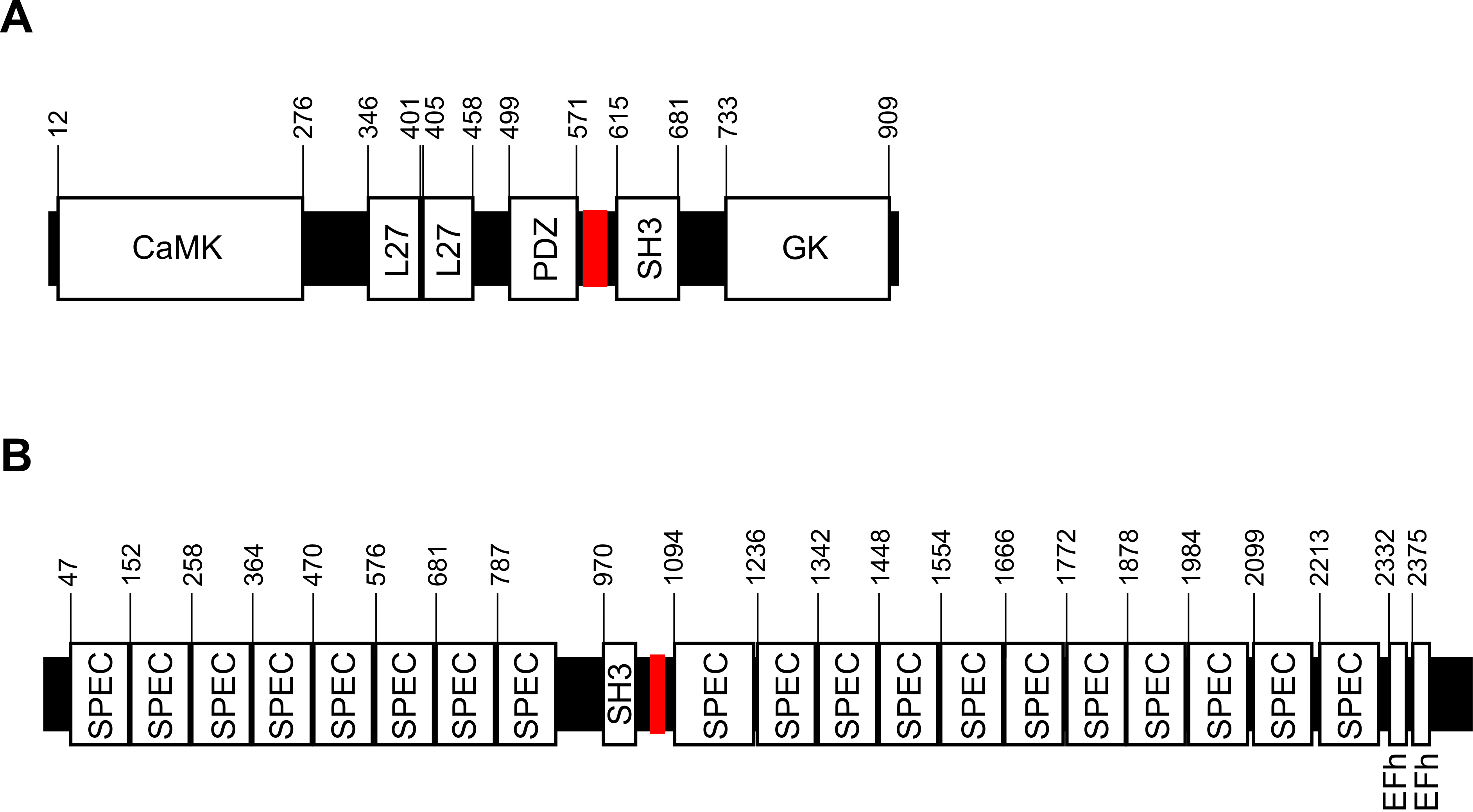

Supplement: Figure S2 — Protein domains of CASK and SPTAN1. (TIF) [file pone.0042697.s002.tif]

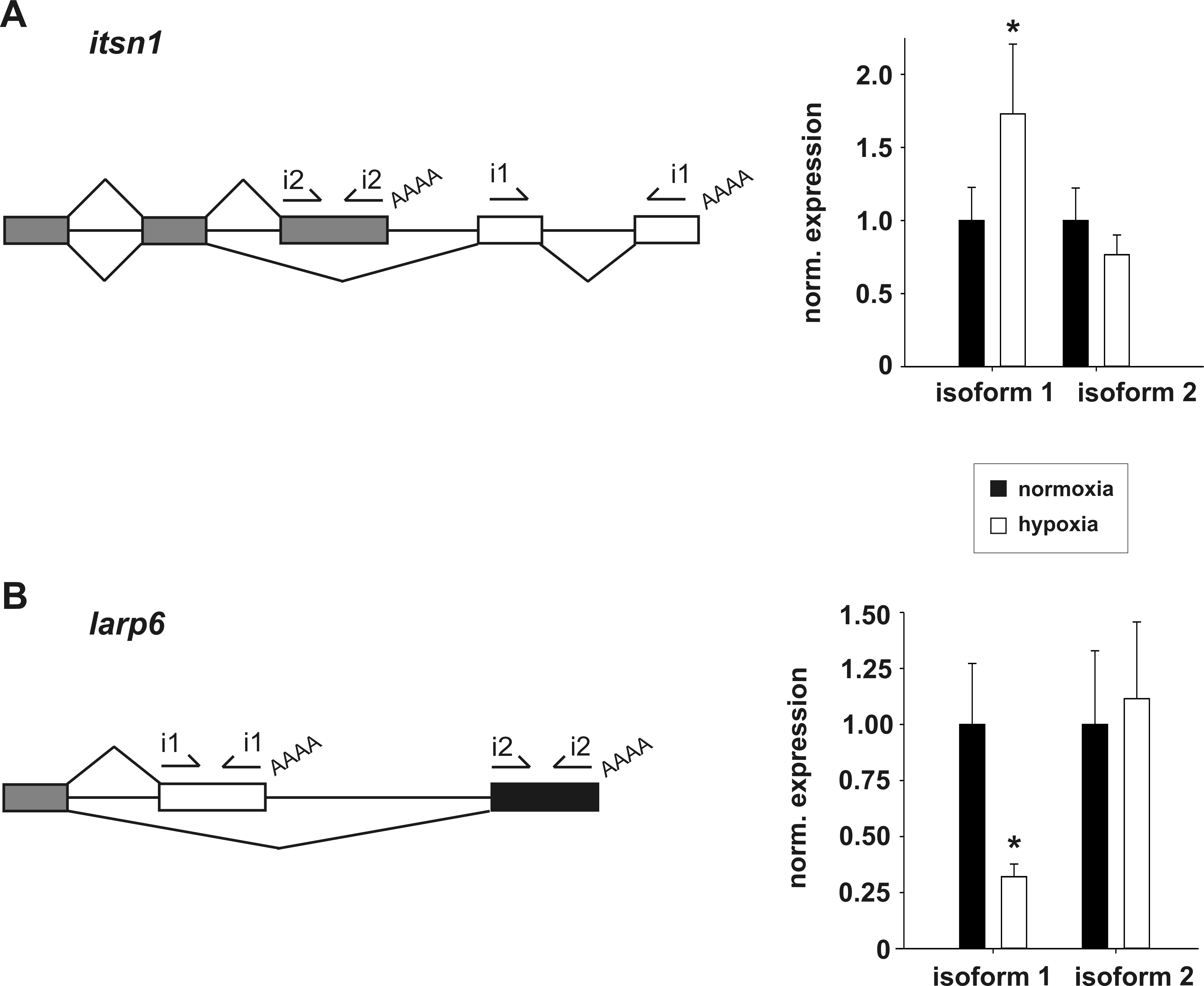

Supplement: Figure S3 — qRT-PCR analysis of APA in itsn1 and larp6 . (TIF) [file pone.0042697.s003.tif]

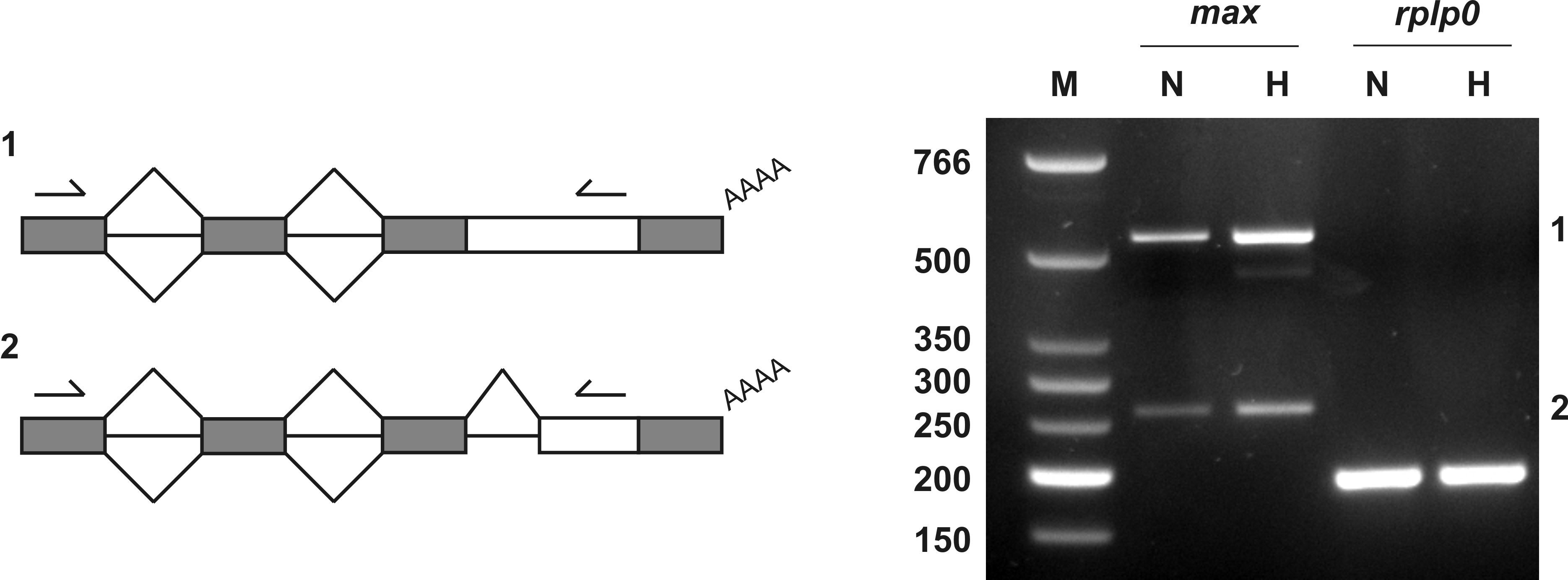

Supplement: Figure S4 — RT-PCR analysis of the intron retention in the max mRNA. (TIF) [file pone.0042697.s004.tif]

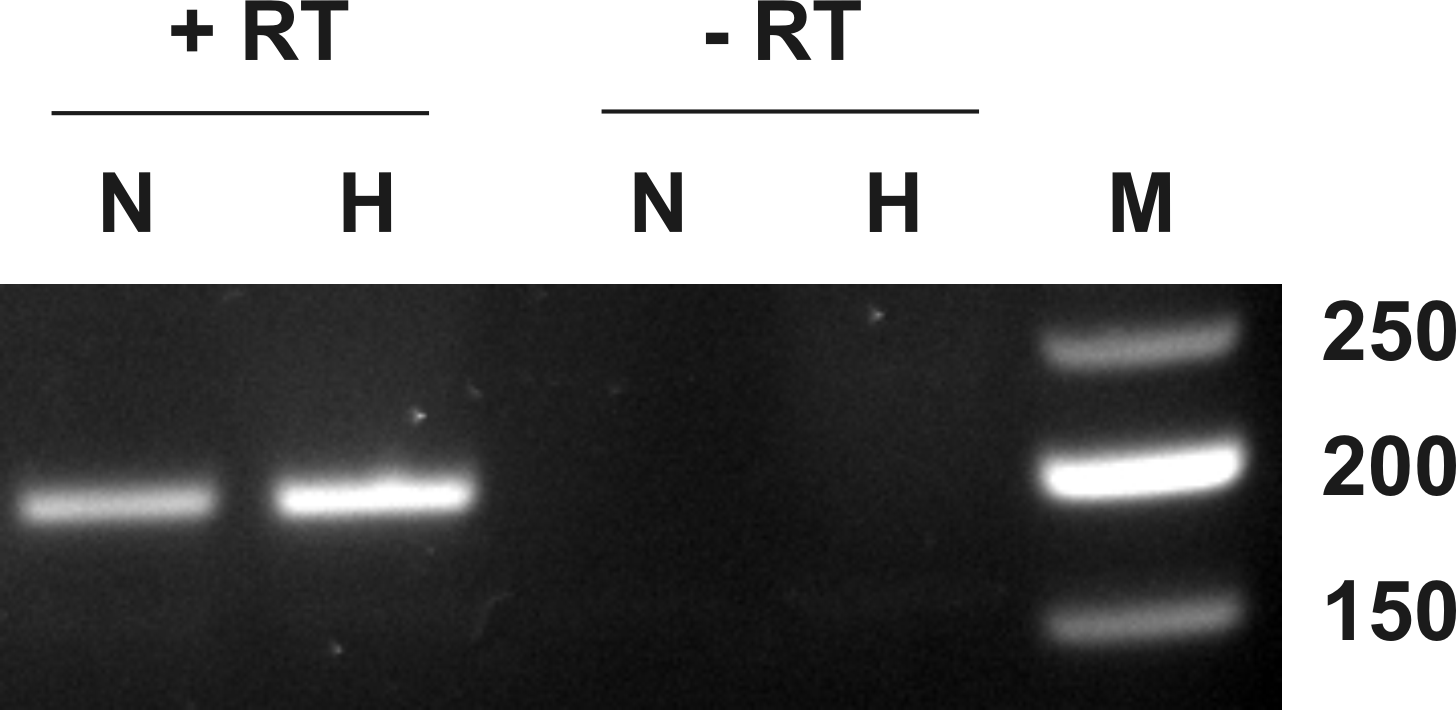

Supplement: Figure S5 — Minus RT control for the intron retention in the max mRNA. (TIF) [file pone.0042697.s005.tif]
