# Supplementary material for: Hypoxia-Induced Alternative Splicing in Endothelial Cells
Source: PLoS One. 2012 Aug 2;7(8):e42697. doi: 10.1371/journal.pone.0042697 (PMC3411717; doi:10.1371/journal.pone.0042697)
Supplement: Table S1 — List of upregulated genes. (PDF) [file pone.0042697.s006.pdf]

Table S1. List of upregulated genes.

Gene symbol	Entrez gene ID	Factor (RMA) [1]	Factor (Iter-PLIER) [2]
acer2	340485	2.64	4.20
adm	133	4.44	4.72
adora2a	135	2.00	2.75
adssl1	122622	5.24	8.06
akap12	9590	2.69	2.89
aldoc	230	3.18	3.58
angptl4	51129	5.50	5.82
ankrd37	353322	4.23	6.11
apln	8862	2.69	3.07
arrdc2	27106	2.25	2.62
bach1	571	2.17	2.53
bhlhe40	8553	3.81	4.72
bnip3	664	2.14	2.20
c13orf15	28984	3.48	3.48
c1orf21	81563	2.50	2.71
chrna1	1134	2.30	4.17
cxcr4	7852	3.68	3.66
cyr61	3491	2.03	2.06
dnah8	1769	2.77	17.75
dusp1	1843	2.19	2.35
dusp6	1848	3.05	3.34
edil3	10085	2.33	2.45
egln3	112399	11.71	96.34
eno2	2026	4.03	4.72
ero1l	30001	2.23	2.23
errfi1	54206	2.03	2.07
fam13a	10144	3.07	4.26
fam189a2	9413	3.97	4.03
fbln5	10516	3.12	3.32
fbxo32	114907	2.33	2.73
galntl2	117248	8.11	7.26
gbe1	2632	2.16	2.20
hk2	3099	2.64	3.68
hsd17b2	3294	3.86	12.47
icam1	3383	2.57	2.77
icoslg	23308	2.08	2.77
igf2	3481	2.17	2.51
inhba	3624	4.79	4.50
insr	3643	2.71	2.93
itga11	22801	2.30	3.20
klhl20	27252	2.36	2.43
klrd1	3824	2.53	3.46
lims2	55679	2.10	4.59
lox	4015	2.50	2.46
maff	23764	2.31	4.14
mertk	10461	2.51	2.64
mxi1	4601	2.25	3.86

myl9	10398	2.11	2.75
ncRNA00204	100132967	4.17	4.11
nedd9	4739	2.53	2.55
nnat	4826	2.41	2.58
nox4	50507	2.17	2.17
npr3	4883	3.66	8.28
p4ha1	5033	3.53	3.43
pde2a	5138	2.41	2.85
pde3a	5139	2.00	2.10
pdgfb	5155	2.19	2.45
pdk1	5163	2.19	2.28
pfkfb3	5209	2.08	2.20
pgf	5228	3.18	3.53
pgm1	5236	2.93	3.39
pim1	5292	2.89	3.92
plac8	51316	3.48	4.06
pparg	5468	5.10	7.46
ppp1r13l	10848	2.36	2.99
ptgis	5740	5.06	5.78
sdc2	6383	2.73	3.78
sdcbp2	27111	2.20	3.94
slc16a3	9123	2.11	2.23
slc25a37	51312	2.06	2.11
slc2a1	6513	5.98	6.41
slc2a3	6515	3.68	3.51
slc6a6	6533	2.14	2.35
slc7a2	6542	2.10	2.31
spry1	10252	2.03	2.17
stc1	6781	10.34	15.03
stc2	8614	2.48	2.93
stx11	8676	2.55	2.68
tagln	6876	2.68	7.41
tgfb1i1	7041	2.06	2.07
timp3	7078	2.62	2.99
tnfsf15	9966	2.00	2.16
tns1	7145	2.04	2.20
vegfa	7422	3.18	3.63
vegfc	7424	2.31	2.66
vldlr	7436	2.53	3.03

[1] Fold induction as predicted by RMA.

[2] Fold induction as predicted by Iter-PLIER.
