# Supplementary material for: Hypoxia-Induced Alternative Splicing in Endothelial Cells
Source: PLoS One. 2012 Aug 2;7(8):e42697. doi: 10.1371/journal.pone.0042697 (PMC3411717; doi:10.1371/journal.pone.0042697)
Supplement: Table S2 — List of downregulated genes. (PDF) [file pone.0042697.s007.pdf]

Table S2. List of downregulated genes.

Gene symbol	Entrez gene ID	Factor (RMA) [1]	Factor (Iter-PLIER) [2]
abcg2	9429	0.44	0.38
acat2	39	0.47	0.39
ace	1636	0.46	0.31
adamts1	9510	0.31	0.21
adamts18	170692	0.43	0.40
adamts9	56999	0.35	0.31
anln	54443	0.32	0.30
apobec3b	9582	0.34	0.35
asf1b	55723	0.21	0.14
aspm	259266	0.35	0.30
aurka	6790	0.41	0.30
aurkb	9212	0.50	0.32
bmper	168667	0.40	0.14
brca1	672	0.41	0.29
brip1	83990	0.37	0.30
bub1	699	0.36	0.35
bub1b	701	0.29	0.26
c12orf48	55010	0.37	0.29
c12orf5	57103	0.36	0.35
c14orf145	145508	0.49	0.46
c7	730	0.45	0.41
casc5	57082	0.31	0.28
ccna2	890	0.38	0.36
ccnb1	891	0.37	0.33
ccnb2	9133	0.34	0.31
ccnd1	595	0.45	0.40
ccne2	9134	0.33	0.30
cdc2	983	0.38	0.36
cdc20	991	0.28	0.27
cdc45l	8318	0.32	0.17
cdc6	990	0.18	0.13
cdca2	157313	0.37	0.25
cdca8	55143	0.40	0.37
cdh11	1009	0.39	0.37
cdkn3	1033	0.46	0.35
cenpe	1062	0.43	0.32
cenpf	1063	0.36	0.33
cenpi	2491	0.33	0.27
cenpk	64105	0.30	0.29
cep55	55165	0.30	0.24
ckap2	26586	0.40	0.39
ckap2l	150468	0.36	0.31
cks2	1164	0.36	0.36
clspn	63967	0.40	0.14
cmah	8418	0.42	0.34
crot	54677	0.45	0.44
cyp1a1	1543	0.47	0.40

depdc1	55635	0.31	0.27
dhcr24	1718	0.22	0.22
dlgap5	9787	0.31	0.27
dpp4	1803	0.39	0.34
dtl	51514	0.18	0.15
eif4h	7458	0.35	0.35
elmod1	55531	0.38	0.13
elovl6	79071	0.31	0.23
entpd1	953	0.41	0.39
ephx1	2052	0.44	0.36
exo1	9156	0.34	0.13
fabp4	2167	0.21	0.21
fam111b	374393	0.17	0.06
fam40b	57464	0.47	0.36
fam83d	81610	0.32	0.26
fancd2	2177	0.42	0.30
fbxo5	26271	0.45	0.40
fen1	2237	0.50	0.37
flrt2	23768	0.39	0.39
foxm1	2305	0.38	0.29
fus	2521	0.45	0.41
gimap7	168537	0.41	0.39
gins1	9837	0.25	0.21
gins2	51659	0.25	0.18
gins3	64785	0.43	0.37
gja4	2701	0.33	0.26
glt8d2	83468	0.35	0.31
gmnn	51053	0.50	0.35
gsdmc	56169	0.48	0.36
gucy1a3	2982	0.39	0.31
hells	3070	0.42	0.34
hist1h1a	3024	0.24	0.22
hist1h1b	3009	0.33	0.30
hist1h2al	8332	0.33	0.32
hist1h2bf	8343	0.48	0.48
hist1h2bm	8342	0.26	0.26
hist1h3a	8350	0.31	0.30
hist1h3b	8358	0.26	0.24
hist1h3c	8352	0.30	0.29
hist1h3h	8357	0.36	0.31
hist1h3j	8356	0.33	0.24
hist2h2ab	317772	0.48	0.47
hjurp	55355	0.49	0.40
hmmr	3161	0.35	0.26
hmox1	3162	0.40	0.31
hnrnpm	4670	0.49	0.36
hspa1a	3303	0.27	0.27
hspa4l	22824	0.44	0.41
hsph1	10808	0.43	0.42
ifit2	3433	0.47	0.36
il1r1	3554	0.37	0.31
KIAA0101	9768	0.32	0.32

KIAA0746	23231	0.44	0.42
KIAA1524	57650	0.37	0.29
kif11	3832	0.28	0.29
kif14	9928	0.47	0.36
kif15	56992	0.40	0.30
kif18a	81930	0.41	0.33
kif20a	10112	0.30	0.28
kif20b	9585	0.42	0.34
kif23	9493	0.40	0.34
kif2c	11004	0.45	0.33
kif4a	24137	0.38	0.31
lrcc17	10234	0.35	0.33
map2k6	5608	0.49	0.34
matn2	4147	0.39	0.38
mcm10	55388	0.33	0.15
mcm4	4173	0.35	0.21
mcm6	4175	0.40	0.38
melk	9833	0.28	0.25
mettl7a	25840	0.24	0.18
mki67	4288	0.29	0.26
mlf1ip	79682	0.35	0.24
mybl2	4605	0.32	0.26
nav3	89795	0.45	0.41
ncapd3	23310	0.45	0.37
ncapg	64151	0.29	0.22
ncaph	23397	0.34	0.24
ndc80	10403	0.32	0.25
neil3	55247	0.46	0.34
nlgn1	22871	0.47	0.43
nme1	4830	0.48	0.45
nqo1	1728	0.22	0.21
nuf2	83540	0.38	0.32
nusap1	51203	0.30	0.30
oas1	4938	0.49	0.31
pbk	55872	0.21	0.20
pcna	5111	0.37	0.26
pde1a	5136	0.43	0.28
pik3c2b	5287	0.47	0.41
pik3r3	8503	0.15	0.14
pla2g4a	5321	0.50	0.48
plk1	5347	0.40	0.36
plk4	10733	0.48	0.42
pole2	5427	0.29	0.21
pop1	10940	0.44	0.30
postn	10631	0.28	0.17
ppfibp2	8495	0.50	0.35
prc1	9055	0.34	0.32
prkcdbp	112464	0.45	0.41
prnd	23627	0.41	0.31
prr11	55771	0.42	0.40
psat1	29968	0.33	0.33
ptpn13	5783	0.39	0.29

pttg1	9232	0.44	0.41
racgap1	29127	0.46	0.38
rad51	5888	0.42	0.34
rad51ap1	10635	0.30	0.22
rfc3	5983	0.37	0.33
rgs4	5999	0.29	0.30
rgs5	8490	0.44	0.40
rrm2	6241	0.28	0.26
sell	6402	0.32	0.15
selp	6403	0.48	0.45
sema6a	57556	0.43	0.39
serpinb2	5055	0.40	0.39
sesn3	143686	0.39	0.37
sgk3	23678	0.49	0.43
sgol1	151648	0.31	0.25
sgol2	151246	0.37	0.28
shcbp1	79801	0.25	0.18
ska1	220134	0.30	0.21
ska3	221150	0.37	0.36
slc40a1	30061	0.41	0.39
slc43a2	124935	0.47	0.32
slc7a11	23657	0.45	0.46
scl7a8	23428	0.39	0.22
slc9a9	285195	0.47	0.25
sort1	6272	0.45	0.40
spag5	10615	0.41	0.31
spc25	57405	0.26	0.20
stat1	6772	0.42	0.44
steap1	26872	0.35	0.31
steap1b	256227	0.45	0.46
steap2	261729	0.35	0.29
stil	6491	0.40	0.37
tacc3	10460	0.48	0.28
tcf19	6941	0.48	0.19
tfec	22797	0.49	0.38
tfrc	7037	0.32	0.34
tk1	7083	0.41	0.23
tmem106c	79022	0.49	0.47
tmem154	201799	0.37	0.13
tmem19	55266	0.49	0.42
tnfsf18	8995	0.41	0.40
top2a	7153	0.33	0.31
tox	9760	0.44	0.42
tpx2	22974	0.30	0.30
trim2	23321	0.48	0.37
trip13	9319	0.37	0.34
tsen15	116461	0.42	0.40
tspan5	10098	0.49	0.48
ttk	7272	0.43	0.33
txnip	10628	0.26	0.28
tyms	7298	0.34	0.33
ube2t	29089	0.37	0.32

ucp2	7351	0.42	0.33
uhrf1	29128	0.24	0.15
wdhd1	11169	0.43	0.38
zwilch	55055	0.48	0.44
zwint	11130	0.30	0.17

[1] Fold reduction as predicted by RMA.

[2] Fold reduction as predicted by Iter-PLIER.
