# Supplementary material for: Hypoxia-Induced Alternative Splicing in Endothelial Cells
Source: PLoS One. 2012 Aug 2;7(8):e42697. doi: 10.1371/journal.pone.0042697 (PMC3411717; doi:10.1371/journal.pone.0042697)
Supplement: Table S3 — List of alternatively expressed probe sets. (PDF) [file pone.0042697.s008.pdf]

Table S3. List of alternatively expressed probe sets.

Affymetrix probe set	Gene symbol	Entrez gene ID	Factor (RMA) [1]	Factor (Iter-PLIER)[2]	splicing event	Predicted outcome
3730694	*ace*	1636	2.68	3.84	APA [3]	NMD [4]
2394641	*acot7*	11332	2.16	3.29	cassette exon	NMD
3927468	*adamts1*	9510	2.53	3.63	internal probe set in first exon	
3700223	*adamts18*	170692	2.08	2.27	internal probe set in first exon	
3554361	*adssl1*	122622	0.23	0.16	first probe set	
2532841	*atg16l1*	55054	2.22	2.22	internal probe set in last exon	
4005893	*cask*	8573	0.43	0.32	cassette exon	alt. protein
2473995	*cenpa*	1058	2.10	4.00	first probe set	
3372287	*cugbp1*	10658	0.42	0.34	APS [5]	alt. N-terminus
3125254	*dlc1*	10395	0.49	0.38	constitutive exon	alt. Protein
3125257	*dlc1*	10395	0.49	0.41	constitutive exon (one of two probe sets)	alt. protein
3850153	*dnmt1*	1786	2.46	2.50	first probe set	
3403018	*eno2*	2026	0.36	0.26	constitutive exon	no AUG [6]
3403020	*eno2*	2026	0.46	0.36	cassette exon	NMD
2610263	*fancd2*	2177	0.28	0.41	constitutive exon	NMD
2367115	*fmo4*	2329	0.44	0.28	internal probe set in last exon	
2732693	*fras1*	80144	0.49	0.34	constitutive exon (one of two probe sets)	NMD
3484601	*fry*	10129	2.50	2.73	constitutive exon	alt. protein
3484607	*fry*	10129	2.10	2.23	constitutive exon	alt. protein
2946384	*hist1h4h*	8365	2.27	2.14	APS (first probe set)	alt. 5'UTR
3848349	*insr*	3643	0.45	0.44	constitutive exon	no AUG
3918909	*itsn1*	6453	2.31	3.12	APA	alt. C-terminus
3980611	*kif4a*	24137	3.51	4.89	internal probe set in last exon	
3631510	*larp6*	55323	0.40	0.33	APA	alt. C-terminus
3631511	*larp6*	55323	0.33	0.28	APA	alt. C-terminus
2575045	*lims2*	55679	0.49	0.24	APS (first probe set)	alt. N-terminus
2669251	*lrrfip2*	9209	0.39	0.34	cassette exon	alt. protein
3568681	*max*	4149	2.30	3.05	intron retention	alt. C-terminus
3568682	*max*	4149	3.63	4.50	intron retention	alt. C-terminus
3568683	*max*	4149	2.66	3.48	intron retention	alt. C-terminus
3568684	*max*	4149	3.32	4.38	intron retention	alt. C-terminus

3568685	*max*	4149	2.68	3.43	intron retention	alt. C-terminus
3568686	*max*	4149	2.66	3.32	intron retention	alt. C-terminus
3235792	*mcm10*	55388	0.28	0.46	constitutive exon	no AUG
2648678	*mme*	4311	0.47	0.41	APS (first probe set)	alt. 5'UTR
2752752	*neil3*	55247	2.28	3.16	constitutive exon (one of two probe sets)	alt. C-terminus
3031738	*nos3*	4846	2.01	2.79	constitutive exon	NMD
3092890	*nrg1*	3084	0.40	0.43	APS (one of three probe sets)	alt. N-terminus
2751119	*palld*	23022	0.50	0.37	constitutive exon	alt. protein
3381233	*pde2a*	5138	0.48	0.40	first probe set	
3811162	*pign*	23556	0.34	0.39	cassette exon	alt. 5'UTR
3908935	*ptgis*	5740	0.30	0.26	last probe set	
3196920	*rfx3*	5991	0.27	0.19	cassette exon	alt. 5'UTR
2683902	*robo1*	6091	0.23	0.25	APS	alt. N-terminus
3108191	*sdc2*	6383	0.46	0.35	last probe set	
2409153	*slc2a1*	6513	0.26	0.21	first probe set	
3373876	*slc43a3*	29015	0.49	0.41	APS (first probe set)	alt. 5'UTR
2699159	*slc9a9*	285195	2.41	4.56	last probe set	
4021157	*smarca1*	6594	0.50	0.41	last probe set	
3190593	*sptan1*	6709	0.44	0.40	cassette exon	alt. protein
2581434	*stam2*	10254	0.29	0.35	internal probe set in last exon	
2411269	*stil*	6491	2.68	2.89	cassette exon or APS (first probe set)	alt. 5'UTR
3908437	*sulf2*	55959	2.48	2.53	first probe set	
3094806	*tacc1*	6867	0.31	0.37	cassette exon	alt. 5'UTR
3776512	*tgif1*	7050	0.43	0.30	internal probe set in first exon	
3597364	*tpm1*	7168	0.41	0.31	APS (one of two probe sets)	alt. N-terminus
3576846	*trip11*	9321	5.82	3.12	cassette exon (one of two probe sets)	alt. protein
3381833	*ucp2*	7351	2.11	2.62	first probe set	
2908181	*vegfa*	7422	0.45	0.40	internal probe set in first exon	
3435119	*wdr66*	144406	0.38	0.30	constitutive exon	NMD
3290223	*zwint*	11130	2.75	4.35	intron retention	alt. 3'UTR

[1] Fold change as predicted by RMA.

[2] Fold change as predicted by Iter-PLIER.

[3] APA = alternative polyadenylation

[4] NMD = nonsense mediated decay

[5] APS = alternative promoter selection

[6] no AUG = the constitutive exon is the first exon of the open reading frame
