# Supplementary material for: Hypoxia-Induced Alternative Splicing in Endothelial Cells
Source: PLoS One. 2012 Aug 2;7(8):e42697. doi: 10.1371/journal.pone.0042697 (PMC3411717; doi:10.1371/journal.pone.0042697)
Supplement: Table S4 — Primer sequences used in this study. (PDF) [file pone.0042697.s009.pdf]

Table S4. Primer sequences used in this study.

Gene symbol	Entrez gene ID	Specificity	fwd-primer 5' → 3'	rev-primer 5' → 3'	Product size [bp]
cask	8573	alt. isoforms [1]	AATGGCATCAGTGT GGCTAAC	ACCTGCAGTTCCAT TTTTGG	324 / 393
cugbp1	10658	isoform 1 [2]	TGTGTTGTGAACTG AGCTTGG	CCACCATCATTTCTG GAAGG	164
cugbp1	10658	total [3]	AGCCCTGCAGACAT TAGCTG	TTGCTGGATACCCG AGTAGG	157
itsn1	6453	isoform 1	GCCAGTTCTTCATCC GAGAC	GTGCAGCAGAAGAC ACTTCG	167
itsn1	6453	isoform 2 [4] 3'UTR	AGCATGGCCACTAT TTTGATG	CTACACGACCACCA GCACAG	147
itsn1	6453	total	CACCCAAAGGCAAG AAATTG	TTCTGCTGAACTTGT TTTAATTGG	162
larp6	55323	isoform 1	AGTTGGAGGACGAG GAGGAG	TGCTGTGCTAGGTG CTGAAG	182
larp6	55323	isoform 1 3'UTR	AATTCAAGGACCAT GCTTGC	GCTGCCTTCAAGGA TCTCAC	171
larp6	55323	isoform 2	AGTTGGAGGACGAG GAGGAG	TCCTCACGCTCGTTC TCAC	151
larp6	55323	isoform 2 3'UTR	AGCATGGCCACTAT TTTGATG	GCCTATCGAGGACA GCTCAG	165
max	4149	isoform 1 intron only	CTGAGTGTTAGAGC CGTCCTG	CACTCCAAGGACCT CAAAGC	196
max	4149	isoform 1 exon/intron	GGGACCACATCAAA GACAGC	CACTCCAAGGACCT CAAAGC	528 / 259
max	4149	total	GGGACCACATCAAA GACAGC	GCTGCTCCAGAAGA GCATTC	186
pign	23556	alt. isoforms	GAGCTGTAGCCTTT GGAGACC	TGTCAAAGATGGAG GCGAAG	185 / 262
pign	23556	isoform 1	TGGCAATTTAATGC TGTTCTTG	TGTCAAAGATGGAG GCGAAG	109
pign	23556	isoform 2	TGATAAACCTGGAA GCTGTTCTTG	TGTCAAAGATGGAG GCGAAG	111
robo1	6709	isoform 1	TTTGGTCATGATATC ACTCCTCAG	TGTATAGCCCAGCG AATTGTC	148
robo1	6709	total	CTCCCCACAGGAAG AACTCC	AGGGGTCCTGAAAT GTAGCC	159
robo1	6709	isoform 2	CATTTGGGGACCCT ATTTCC	GACGGGAGCCTGAA CAGAG	132
sptan1	7168	alt. isoforms	GAACGATCGTCAGG GTTTTG	TACGCTTCTCACCC AGTTCC	164 / 224
tpm1	6091	isoform 1	GCTGGACCACGAGA GGAAG	TCTCATCTGCTGCCT TCTCAG	158
tpm1	6091	total	TCCAGCTGGTTGAG GAAGAG	ATGTGCTTGGCCTCT TTCAG	187
rplp0	6175	total	TCGACAATGGCAGC ATCTAC	ATCCGTCTCCACAG ACAAGG	191
ucp2	7351	5' end	GCCTTGGGATTGAC TGTCC	GAGCCGCAGGGAGA ACAC	100

ucp2	7351	total	AAAGCACCGTCAATGCCTAC	CGATGACAGTGGTGCAGAAG	225
vegfa	7422	total	CCCTGATGAGATCGAGTACA	AGCAAGGCCCACAGGGATTT	245

[1] Primers frame a cassette exon and amplify both isoforms.

[2] Primers detect only the isoform predicted to change its expression under hypoxic conditions.

[3] Primers detect (nearly) all predicted isoforms of the gene.

[4] Primers detect an isoform not predicted to change its expression under hypoxic conditions.
